# Alternative community-led intervention to improve uptake of cataract surgery services in rural Tanzania—The Dodoma Community Cataract Acceptance Trial (DoCCAT): a protocol for intervention co-designing and implementation in a cluster-randomized controlled trial

**DOI:** 10.1093/biomethods/bpae016

**Published:** 2024-03-08

**Authors:** Frank Sandi, Gareth Mercer, Robert Geneau, Kenneth Bassett, Deogratius Bintabara, Albino Kalolo

**Affiliations:** Department of Ophthalmology, The University of Dodoma Medical School, Dodoma, Tanzania; Department of Ophthalmology & Visual Sciences, University of Toronto, Toronto, Canada; Division of Ophthalmology, University of Cape Town, Cape Town, South Africa; Department of Ophthalmology, The University of British Columbia, Vancouver, Canada; Department of Community Medicine, The University of Dodoma Medical School, Dodoma, Tanzania; Department of Public Health, St Francis University College of Health and Allied Sciences, Morogoro, Tanzania

**Keywords:** Community-led Intervention, Uptake Cataract services, cRCT, cataract surgery, Rural Tanzania

## Abstract

Age-related lens opacification (cataract) remains the leading cause of visual impairment and blindness worldwide. In low- and middle-income countries, utilization of cataract surgical services is often limited despite community-based outreach programmes. Community-led research, whereby researchers and community members collaboratively co-design intervention is an approach that ensures the interventions are locally relevant and that their implementation is feasible and socially accepted in the targeted contexts. Community-led interventions have the potential to increase cataract surgery uptake if done appropriately.

In this study, once the intervention is co-designed it will be implemented through a cluster-randomized controlled trial (cRCT) with ward as a unit of randomization.

This study will utilise both the qualitative methods for co-designing the intervention and the quantitative methods for effective assessment of the developed community-led intervention through a cRCT in 80 rural wards of Dodoma region, Tanzania (40 Intervention). The ‘intervention package’ will be developed through participatory community meetings and ongoing evaluation and modification of the intervention based on its impact on service utilization. Leask’s four stages of intervention co-creation will guide the development within Rifkin’s CHOICE framework. The primary outcomes are two: the number of patients attending eye disease screening camps, and the number of patients accepting cataract surgery. NVivo version 12 will be used for qualitative data analysis and Stata version 12 for quantitative data. Independent and paired t-tests will be performed to make comparisons between and within groups. P-values less than 0.05 will be considered statistically significant.

## Introduction

The recent released WHO data have shown there are at least 2.2 billion people worldwide who are visually impaired. In at least 1 billion cases, the problem could have been prevented or has yet to be addressed, particularly cataract [1, 2].

In all world regions, women bear most of the blindness and vision impairment at 55%. This is largely due to social factors where women do not get to access surgical services with the same frequency as men [1–3]. Several studies have shown that the cataract surgical coverage among women in Sub-Saharan Africa (SSA) and South Asia is nearly always lower, sometimes only half that in men [4–6].

The barriers to utilizing cataract surgical services in SSA are well known: lack of treatment awareness, fear of surgery, poor geographical access, and inability to pay for direct and indirect costs [5, 7–9]. However, there is still limited evidence about what works to mitigate these barriers and factors associated with low utilization.

There is some observational evidence that bringing eye health services closer to community, coupled with other health system interventions, have led to an increase in the volume of cataract surgeries in countries and districts targeted by Vision 2020: right to sight programme [10, 11]. Most of this evidence comes from studies that applied before-after designs thus posing several limitations in generating rigorous evidence and generalization of findings [6, 12–14].

Recent randomized control trials and systematic reviews, while finding little evidence for non-community-led interventions to improve access to cataract surgery, did find evidence that community-led interventions have the strongest chance of improving health seeking behaviour at the community level [12, 13, 15]. In a community-led intervention, a community identifies its own needs and mobilizes itself to respond to those needs [16, 17].

In this study, the process for the development of the community-led intervention will be based on the recently suggested circle by Leask *et al*. in which they described four stages: Planning (framing the aim of the intervention), Conducting (Defining the intervention and co-creation), Evaluating (the process and the effectiveness through the cluster-randomized controlled trial (cRCT) in this case), and Scaling (of the findings and possibility of scaling up the intervention) [18]. The aim is to produce community-led health interventions that are more effective, efficient, and sustainable than those of traditional investigator-led trials [15, 19, 20]. This model has proven to be effective in controlling Dengue virus transmission in Mexico and improving nutrition status in several Tanzanian communities and has been advised by various studies as best means to address some of the complex public health issues by tailoring interventions [21–24]. However, this study will be the first of its kind in the field of eye health services research aiming at co-developing, implementing, and evaluating the impact of a ‘community-led intervention package’ for increasing cataract surgery uptake in rural Tanzania among adults of 50 years and above.

The Leask *et al*. four stages will be guided by Rifkin’s CHOICE Framework. This framework is a tool to evaluate and plan community-led health interventions that aim to promote equity and empowerment. The framework is based on the work of Amartya Sen, a Nobel laureate economist and philosopher [25, 26]. The framework uses the acronym CHOICE to describe six criteria that distinguish between target-oriented and empowerment approaches to participation. The criteria are: **C**ontrol over resources—while carrying out this trial decisions on how to allocate and use resources for the intervention should be discussed by the community and agreement reached. **H**ealth improvement—explaining to the community the expected health outcomes of the intervention and how are they will be measured. **O**rganizational characteristics—how is the community need to be organized for the intervention will be discussed. **I**nstitutionalization—how will the intervention be integrated into the existing health system and sustained over time, need to be discussed at every stage. **C**apacity building—skills and knowledge of the community members and community health workers enhanced by the intervention implementation. **E**nabling environment—Leadership, communication, and ethical issues need to be addressed during the co-created intervention.

The primary objectives are (i) to co-design a community-led intervention for increasing the uptake of eye health services, including presentation at screening camps and accepting cataract surgery and (ii) to implement the community-led intervention through a cRCT and assess its effectiveness and explore how implementation-related factors influence the achievement of the desired outcomes in different sociocultural settings of Dodoma region, Tanzania.

## Methods and analysis

### Study area

The study will be conducted in Dodoma region of Tanzania. Dodoma is administratively divided into eight district councils. Currently, there are a total of 3.08 million people, 209 wards and people aged 50 years and above account for approximately 10% of the population [27]. Dodoma is one of the regions with the highest prevalence of cataract blindness at 17.7%, which is above the national average of 15% [28]. To address eye health care needs, the regional eye care team has established a regular eye outreach programme since 2017. This justify and favours the settings of this trial to be done in Dodoma region, as there is ongoing community eye outreach programmes (CEOP) aiming at screening patients at their communities. These were initiated by the regional health committee and they have not shown to be very promising in increasing cataract services uptake and henceforth this community-led trial is designed. The current CEOP is done twice each month by a team of assistant ophthalmic medical officers, optometrist, ophthalmic nursing officer, and a Programme coordinator. The main activities entail screening for refractive errors and simple eye diseases by use of a pen torch. Those needing further management and/or surgery are listed down and referred to a base hospital.

### Overall study design

This protocol paper is structured conforming to the Standard Protocol Items: Recommendations for Intervention Trials (SPIRIT) 2013 Checklist [29]. The project will be having two components as (i) Intervention co-creation component using participatory action research approaches [i.e. Focus Group Discussions (FGDs), and Key Informant Interviews (KIIs)] (ii) Assessment of effectiveness and scaling of the intervention through a cRCT. A community-led participatory action research design was chosen because the intervention development and implementation will largely be locally designed and adapted by communities. A community-led participatory action research study design offers an ideal opportunity to engage the community at every stage to develop their own intervention package and implemented for assessment of effectiveness of the interventions, the role of implementation factors, and strategies facilitating the sustainability and scaling up of the intervention package. The cRCT was chosen because it avoids the selection biases and provides a robust way of getting strong correlation of study variables. In this setting, a cluster is defined as an administrative ward which usually has a typical population ranging from 8000 to 12,000.

### Study component I: Community-led intervention co-creation

#### Study design and co-creation process

This component involves a qualitative participatory approach, mainly for the intervention co-development process using the four stages (Planning, Conducting, Evaluating, and Scaling) of participatory action research methods.

**Figure 1.**
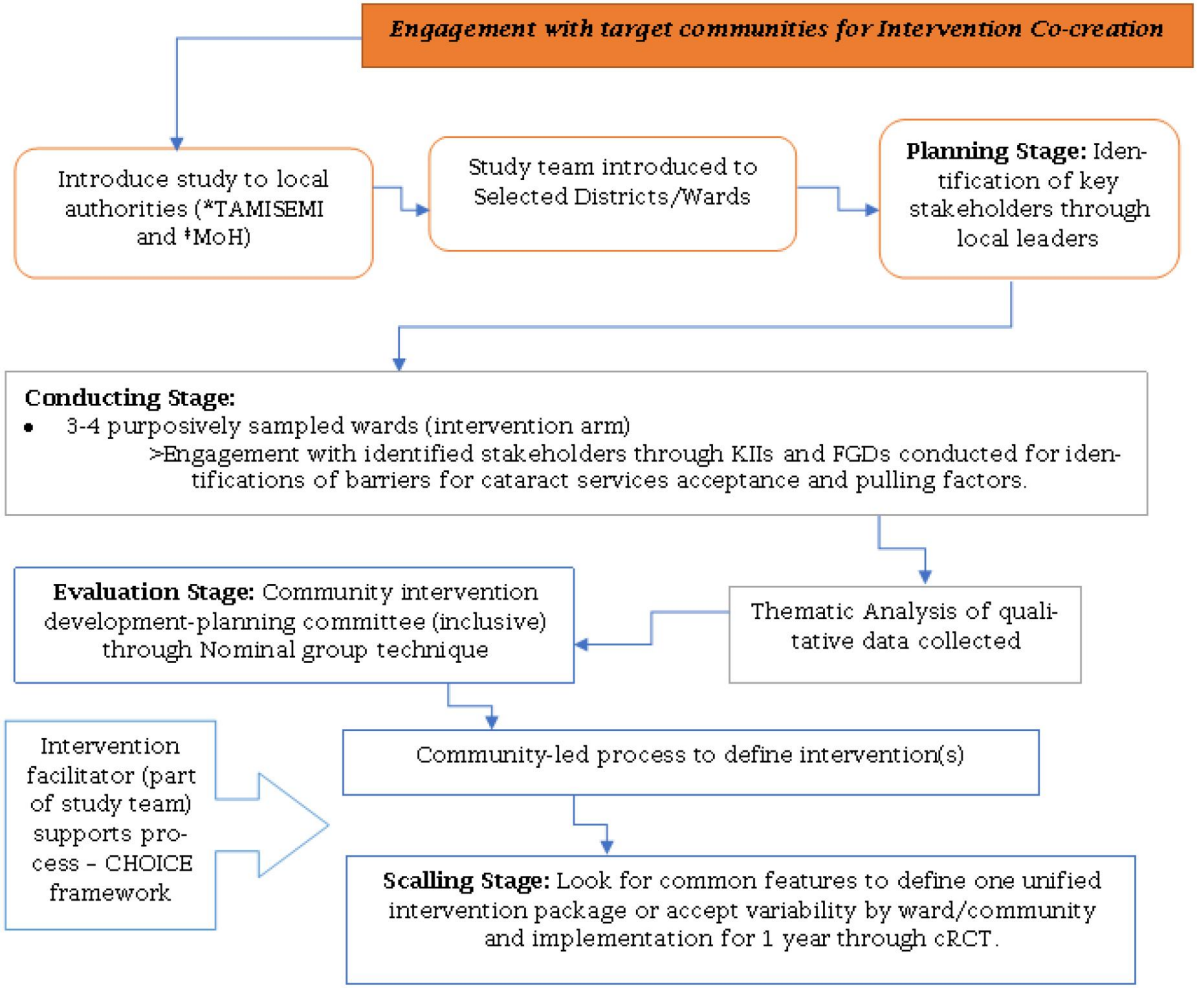
Community engagement and intervention co-creation flow diagram.*^a^TAMISEMI—regional and local authorities, ^b^MoH—Ministry of Health, Tanzania*

- **Planning Stage:**
  - Objective: This stage will entail framing of the aim of the study together with the district and ward leaders so as to get the participants for next stage.
  - Participants: Leaders of Selected pilot 3–4 wards including local health facility in-charge, District eye care coordinators and other first-hand informants of eye health in the ward. As a first step, the research team will meet leaders of selected wards to explain the community-led intervention design process (CLIDP). They will be asked for their assistance in identifying key stakeholders for involvement in the CLIDP through the open community meeting as outlined in Fig. 1
  - Sampling: Leaders of the respective wards will be requested to identify stakeholders for the community-led intervention design process.
  - Methods: Qualitative techniques entailing secondary data review to obtain the contact of the ward leaders so as to engage onto this stage together with community open meetings to identify stakeholders for next stage.
- **Conducting stage**
  - Objectives: The research team will be interested in finding out the perceived levels of cataract visual impairment needing surgery, and the factors that act as facilitators or barriers for accepting cataract surgery.
  - Participants: Identified stakeholders of the concerned wards will be engaged in this stage.
  - Sampling: Representatives of the identified community groups within each ward which include beneficiaries of eye health services, women’s group representatives, youth and religious leaders, community leaders/elders, and health facility in charge
  - Methods: This will entail open community meetings, FGDs, and KIIs of the above groups. The Study team will be led by social scientist who will be providing the technical aspect of data collection in this stage together with the study coordinator and an ophthalmologist. Community representative upskilling will be done prior to the engagement; it entails training on the purpose, process, and expectations of co-creation of the intervention through the use of FGD and KII. This will include explanation of role of the facilitator, the importance of active listening, the types of questions and responses, aim of co-creation and the ethical principles of confidentiality and respect. Further community upskilling will be provided as need arise. For feasibility reasons, the intervention development component will involve a small sub-sample of 3–4 intervention wards (purposefully sampled), and a total of 12–15 FGDs and 6–8 KII are planned to be conducted. The development of interview questions will be guided by findings from a recent RAAB Study in nearby regions of Tanzania [4, 30–32].
- **Evaluating Stage**
  - Objective: Community-led co-creation of the intervention through community feedback and consensus on the agreed components of the intervention.
  - Participants: Members of the initially engaged communities and technical group of CHMT members at the district level.
  - Methods: After the initial data collected, they will be analysed using NVivo version 12 software for coding and for exploring outcome patterns and configurations of factors associated with the study outcomes. Once specific themes are obtained, the study team will present the findings back to communities to support the actual development of the intervention package. Conducting of the co-creation process through community feedback and nominal group techniques (NGTs) to manifest ownership and defining the intervention for implementation. It will be a free flow of ideas from the community member’s feedback and applicability of the co-created intervention will be put in the Rifkin’s CHOICE framework by the study team, along with appointed members of the community who will form a steering committee for the intervention. Iteration and refining of the intervention will be done through discussion, troubleshooting and feedback in the piloted wards before the scaling up to all the intervention wards. The NGT will be done through recording on a flipchart of main barriers and solutions from the conducting stage then discuss through them with the community members for priority setting.
- **Scaling Stage**
  - The agreed local intervention will be scaled up in the rest of the intervention wards through the study component II. There are various models of scaling up but in our study we will use generalizable model in which the sampled local stakeholders will be representing the wider wards in which the intervention will be utilized. Once the planning committees of the selected communities have designed and established the details of their local interventions, a comparison across them to identify common elements will be done. The creation of a unified ‘intervention package’ to be used for all remaining intervention communities will follow.

### Study component II: Effectiveness of the intervention implementation

#### Study design and target population

The second study component will be a two-armed, pragmatic, cRCT to estimate the effectiveness of the community-developed intervention implementation. The clusters in this case are administrative rural wards of Dodoma region. The study will be pragmatic in the sense participants and data collectors will not be masked to the allocation status of the ward, and no attempts will be made to prevent ‘contamination’ of control wards by research activities conducted in intervention wards, thereby allowing estimation of a more ‘real-world’ effectiveness of the intervention [33].

**Figure 2:**
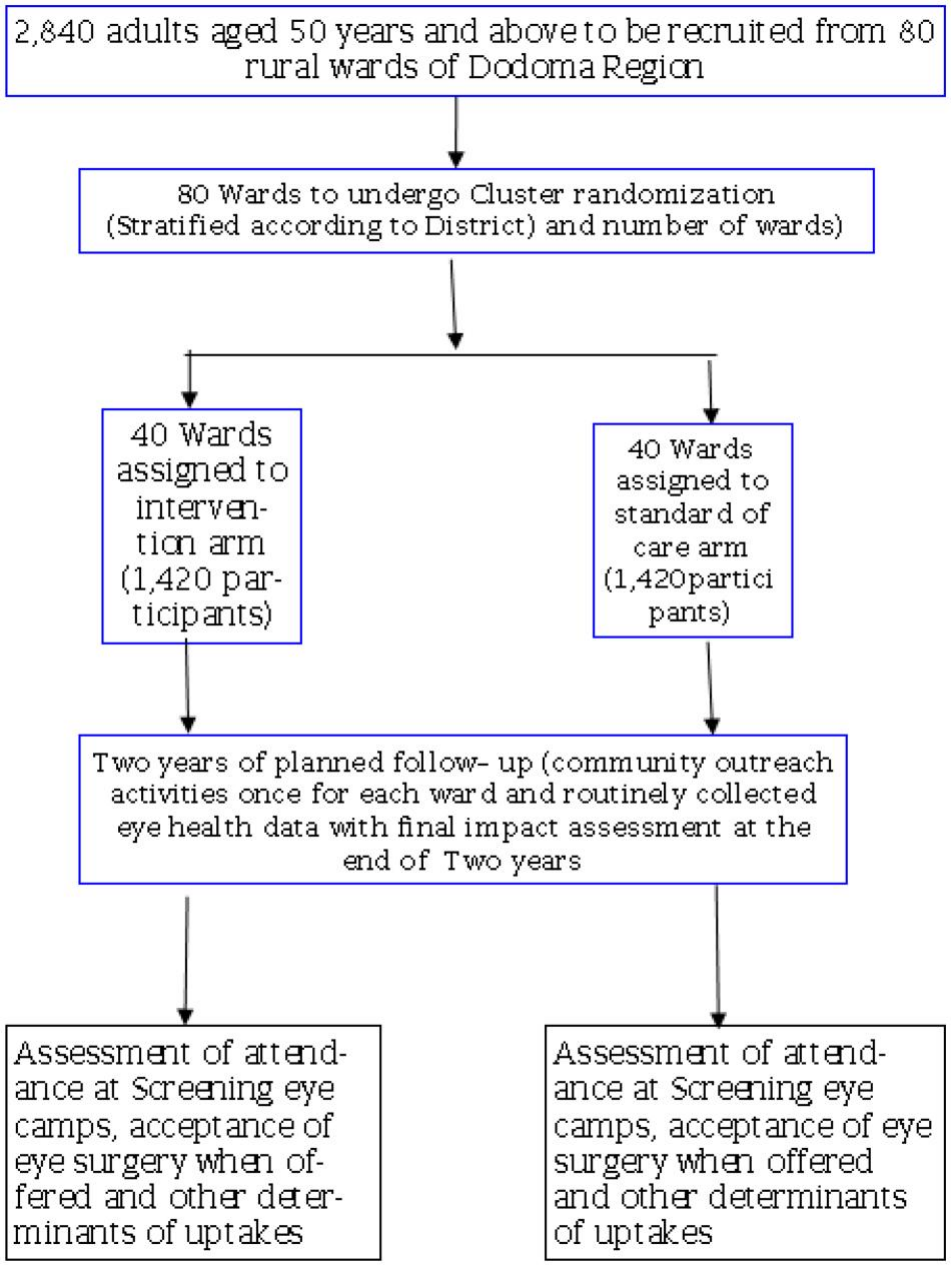
Study flow for schedule of enrolment, interventions, and assessment

The primary focus of this portion of the study is the effectiveness of the community-led interventions in increasing uptake of eye health screening services and cataract surgery. Figure 2 explains the study flow for schedule of enrolment, interventions, and assessment.

### Study population and follow-up

Using data from the 2022 Tanzanian national census (accounting for predicted annual population growth of 2.7% up to the year 2023 for Dodoma region), we expect an average of 1420 adults aged 50 years and above in each study arm (coefficient of variation = 0.4).

Two years of follow-up will yield a mean of 2840 person-years of observation per intervention arm. Based on unpublished findings from a pilot study involving four wards conducted in 2020 by our research team we anticipate a screening rate (i.e. rate of presentation to screening camps among population >50 years of age) of 0.198 per person-year in control villages with an inter-village coefficient of variation (k) of 0.4. For cataract surgery, we anticipate a rate of 0.045 per person-year in control villages based on an estimated prevalence of visually significant cataract of 17.7%, and cataract surgical coverage of 25%. We conservatively assumed we would observe a level of inter-ward variation in surgery acceptance rates like that for screening rates (k = 0.4).

### Sample size, randomization, and allocation concealment

Using the sample size calculation method for unmatched cRCTs described by Hayes and Bennett [34], it is calculated that 40 wards will need to be enrolled in each study arm to achieve 80% power to detect a minimum 30% increase in screening examination and cataract surgery acceptance rates associated with the intervention at the 95% confidence level.

Population data from the 2022 Tanzanian national census (accounting for predicted population growth up to 2023) will be used, we expect an average of 1420 adults aged over 50 years in each study ward (coefficient of variation = 0.43).

The sample size calculation method for unmatched cluster-randomized trials with rate outcomes described by Hayes and Bennett was used:

c=1+Zα/2+Zβ2λ0+λ1y+k2λ02+λ12/λ0-λ12

where c is the number of clusters, $Z_{\alpha /2}$ is the standard normal distribution based on level of significance (1.96), $Z_{\beta }$ is the standard normal distribution value based on the power of $100\left(1-\beta \right)\%$ (using 80% power $Z_{\beta }=0.84)$, $\lambda_{0\;}$ and $\lambda_{1}$ rates of the outcome in absence and presence of intervention, respectively, y is the person-years of follow-up in each group ward, and k = 0.4 is the coefficient of variation of the population rates between wards within each group. To detect a minimum 30% difference in outcome rates comparing intervention to control wards, we calculate that we will need to enrol 39 wards in each study arm for the presentation at screening camps outcome (outcome 1), and 42 wards in each arm for the cataract surgery outcome (outcome 2). To sufficiently power the study for both outcomes, we will sample a total of 40 wards in each study arm taking into account the design effect as well.

#### Intervention wards

In addition to the standard routine cataract screening services, there will be implementation of the co-developed intervention package, which will continue for two years. Screening camps will be done once per year for each cluster. Wards used for co-creation will also be included as part of intervention wards.

#### Control wards

Control wards will receive the standard community eye screening routine package as it has been carried out, at a frequency of once per year. A comparison of the two arms is shown in Table 1.

**Table 1.** Comparison of the two arms of the study.

Item	Intervention arm	Control arm
Community sensitization	Yes	
	Local announcements method available: posters and announcement in churches, mosques, and schools.	
	*Additional community-determined sensitization methods*	N/A
Provision of eye diseases screening camp at a ward	A team composed of ophthalmologist/cataract surgeon, two ophthalmic nurses, optometrist, and outreach coordinator	
	*Intervention facilitator for continued intervention implementation*	NA
Referral from ward to base hospital for surgery	Yes, with the standard usual support	
	*Additional community-determined support to attend base hospital*	NA
Assessment of primary outcome	Same for both arms (trained field worker)	
Assessment of referrals	Outreach team	
	*Study coordinators follow up for non-attenders*	
Post-op care	Standard care as usual	
	*Qualitative study on reason for non-attendance*	NA

### Eligibility and recruitment

#### Clusters/wards/individuals

All rural wards of Dodoma region are eligible to be selected. A total of 80 wards will be selected at random from the administrative list of all wards and randomized 1:1 to control and intervention arms. All adults 50 years of age or older who attend community eye outreach camps will be enrolled.

### Data collection procedure

During the 2-year period from the start of the intervention, community eye camps will be offered once per year in each study ward. At screening camps, attendees will have their address documented, standardized ophthalmic examinations performed (visual acuity testing using a Snellen tumbling E chart, pinhole for those worse than 6/18, direct ophthalmoscope (arc light), and portable slit lamp examinations)

All individuals found at screening camps to have operable cataract (defined as BCVA worse than 6/36 in the better Seeing Eye with cataract as the probable primary cause for visual impairment) will be counselled about the option for surgery and given a written referral to the base hospital. Additional instructions and assistance will be given as per the co-created intervention package in the intervention arm. Community health workers (community eye champions) diaries will also be used to monitor implementation of the intervention and any other observation during implementation.

## Study masking

Community-led interventions are difficult to blind since the wards are already classified into controls and intervention. However, the study statistician and the data clerks at the base hospital will be masked as to whether the patient comes from an intervention or control ward.

## Data collection tools

All clinical data collected at screening camps will be captured in a standardized fashion, in real time using portable electronic devices running the CTO Survey data collection mobile app. The data will be backed up to a central, secure database at the end of each day. The database will be used to track individuals from study villages who were referred for cataract surgery and progression of implementation.

## Description of variables

### Outcomes variables

#### Primary outcome

The primary outcome will be the proportion of all eligible persons with operable cataract who accepted and show up for cataract surgery at the base hospital overall and stratified by age, sex, and VA levels. Based on the 2022 population census, the appropriate population of people aged 50 years and above will be sought from the ward authorities for denominator calculation.

#### Secondary outcomes

- Proportion of all eligible persons attending at outreach eye examinations sites overall and stratified by age, sex, and VA levels (Screening rates)
- Acceptability, appropriateness, and fidelity of the community-led intervention. At study outset, distance of each study ward from the base hospital will be measured. Social economic status of each ward, Education levels, and economic activities of each ward will be obtained from the recent released National censors results.
  - *Acceptability* of the intervention will be assessed by using the standardized Acceptability of Intervention Measure questionnaires to evaluate the affective attitude, burden, perceived effectiveness, and ethicality of an intervention [35, 36].
  - *Appropriateness* will be assessed by using the Intervention Appropriateness Measure questionnaires to measure the perceived need, suitability, alignment, or compatibility of the intervention with the context, culture, or priorities of the participants and the research team [36].
  - *Fidelity* which is the extent to which the intervention is delivered or implemented as intended by community will be assessed using the Fidelity of Implementation Rating System checklist that evaluates the adherence, quality, and differentiation of an intervention delivery [37, 38].

## Statistical analysis and data management

Analysis for baseline characteristics will be through descriptive statistics and level of significance at 0.05% will be considered significance. Results will be analysed by Intention-to-treat analysis in which clusters are analysed as to their group which were randomized prior will be used to determine the mean differences between arms using t-tests or Wilcoxon test (as appropriate) and mixed effects regression modelling of cataract surgery acceptance rate will be done so as to account for clustering at the ward level. Results will be reported as the difference in primary and secondary outcome between the intervention and control arms. We will conduct sensitivity analyses controlling for any imbalances in remaining baseline characteristic at participant and cluster level. Because mixed effects models are robust to data missing at random (MAR), we will assess data missingness. If missing data are not MAR, we will conduct multilevel multiple imputation.

Further quantitative descriptive analyses will be used to provide an understanding of the trial results that require further explanation (e.g. control clusters that demonstrate substantial improvement in outcomes, despite not receiving intervention, and reason for not attending surgery).

The trial will be reported using the 2012 CONSORT guidelines, with the updated cRCT extension [39].

## Coordinating centre

The research team at the University of Dodoma will provide on-site support and implementation of the study.

## Monitoring

Institutions offering ethical clearance and local government authorities will be monitoring the study closely. There will also be an advisory committee composed of local expert and specialists in this field. Data will be collected through use of portable electronic devises, which will be securely backed up and cleaned on daily basis. We do not plan to perform an interim data analysis of the primary outcome. But maybe we will do an interim safety analysis after the first year of the cRTC looking at rates of adverse surgical outcomes with a plan to pause the trial and evaluate the causes if these rates are substantially higher than the current average in Tanzania.

## Participant and public involvement statement

This community-led intervention trial offers a maximum involvement of the community and participants. Community perceptions of the intervention will be determined from time to time and if anything is not working well then mitigation measures will be set in place to make sure the study is implemented as expected by the community. Results of the intervention implementation process will be shared through quarterly wards meetings, which all community members will be invited to attend, and where they will be able to make recommendations for refinement of the intervention package.

## Ethics and dissemination

The study protocol has received ethical clearance from National Institute for Medical Research of Tanzania and the University of Cape Town.

We aim to publish the initial qualitative data on co-creation process and end project progress papers in peer-reviewed scientific articles in open access journals.

## Discussion

This community-led trial is intended to develop and implement a ‘community-led intervention package’ for increasing cataract surgery uptake in rural Tanzania.

Dodoma is one of the regions in Tanzania with a high prevalence of cataract blindness. It has also been among the hardest hit by trachoma due to water scarcity. It is believed that the intervention package developed by the community might address these additional ocular conditions by improving presentation to community eye health outreach camps.

The IAPB and WHO target to eliminate avoidable blindness has come to an end and been updated to the concept of integrated people-centred eye care, which this study is built upon [40]. This study is novel in using a community-led participatory action research approach for development of the intervention and a cluster-randomized trial methodology for rigorously assessing if the package of community-led intervention(s) will bring about the desired changes. If the results of the trial are positive, a plan for scaling up to other rural areas will be devised.

## Data Availability

There are no data collected for now, but the randomization process can be made available if requested. Data will be made available at Open Science Framework (OSF- osf.io/48jmq) research management and collaboration tool.
